# Relationship between sleep duration and sociodemographic characteristics, mental health and chronic diseases in individuals aged from 18 to 85 years old in Guangdong province in China: a population-based cross-sectional study

**DOI:** 10.1186/s12888-020-02866-9

**Published:** 2020-09-16

**Authors:** Xie Chen, Shi-Bin Wang, Xue-Li Li, Zhuo-Hui Huang, Wen-Yan Tan, Hai-Cheng Lin, Cai-Lan Hou, Fu-Jun Jia

**Affiliations:** 1 Guangdong Mental Health Center, Guangdong Provincial People’s Hospital, Guangdong Academy of Medical Sciences, No.123, Huifu Xi Road, Guangzhou City, 510000 Guangdong Province China; 2 Medical College of Shantou University, No.22, Jinling Road, Shantou City, 515041 Guangdong Province China; 3 Affiliated School of Medicine of South China University of Technology, No. 381, Wushan Road, Guangzhou City, 510000 Guangdong Province China

**Keywords:** Sleep duration, Chronic diseases, Mental health, Population-based study

## Abstract

**Background:**

Sleep is vital for maintaining individual’s physical and mental health. Prior studies have reported close relationships between sleep duration and chronic diseases. However, in China, the prevalence of aberrant sleep duration and the associations between sleep duration and chronic conditions still merit studying in Guangdong province. This study aimed at examining the relationship between sleep duration and multiple dimensions of sociodemographic characteristics, mental health and chronic diseases in Guangdong province in China, with a large population-based data of individuals aged from 18 to 85 years old.

**Methods:**

This study aimed at analyzing the sociodemographic and clinical characteristics of the population in Guangdong province. Multistage stratified cluster sampling was applied for this study. 13,768 participants from Guangdong province were interviewed with standardized assessment tools, including Patient Health Questionnaire-9 (PHQ-9) and Generalized Anxiety Disorder (GAD-7). Basic socio-demographic information, mental health and chronic diseases information were collected. Self-reported sleep duration was classified as three types: short (< 7 h), normative (7-9 h) and long (≥9 h).

**Results:**

The mean sleep duration was 6.75 ± 1.11 h. Short sleepers had a higher prevalence of chronic diseases, including anemia (6.2%, *p =* 0.024), gout (2.8%, *p =* 0.010), hyperlipidemia (3.9%, *p* = 0.003) and low back pain (5.6%, *p* = 0.020) than other types of sleeper. Multinomial logistic regression analysis revealed that short sleepers were more likely to have low income level, have depressive symptoms, be ex- or current drinkers and be overweight. Anemia, hyperlipidemia and low back pain were all risk factors for short sleep, while malignant tumor was risky for long sleep.

**Conclusions:**

Low income level, drinking status, being overweight, and chronic conditions may be associated with aberrant sleep duration in Guangdong province general population. Short sleepers have a higher risk of suffering from anemia, hyperlipidemia, and low back pain, while long sleepers are more likely to have malignant tumor. Health professionals should value the sleep patterns in general health care and attach importance to conduct further epidemiologic surveys to explore the relationship between sleep duration and health.

## Background

Sleep is vital for maintaining individuals’ homeostasis. Previous studies have mentioned that people with aberrant sleep duration – meaning not getting proper sleep time each night, have a higher risk of chronic comorbidities, including cardiovascular disease [1], cerebrovascular disease [2], hyperglycemia [3], hypertension [4], obesity [5], anemia [6], and respiratory disease [7]. Comorbidities of sleep problem have aroused people’s attention. Short and long sleep duration have been suggested to be risk factors for chronic diseases [8]. Insomnia problem is a common cause of short sleep duration. People with insomnia problem may find it hard to fall asleep or maintain asleep, and feel tired during daytime activities [9]. Based on one of the mechanisms of insomnia, short sleep duration has been considered to be associated with activation of the hypothalamic-pituitary-adrenal (HPA) axis [10]. Multiple metabolic processes through the regulation of HPA axis could be affected. Changes in HPA axis function may negatively affect the cardiovascular system, leading to hypertension, atherosclerotic plaque formation, insulin resistance, dyslipidemia, and central obesity [11].

Over the past three decades, China has experienced rapid socioeconomic and lifestyle changes, leading to a number of public health problems including increasing smoking and alcohol use, high calorie diet, lack of physical exercise, electronic screen exposure, and increased prevalence of chronic diseases and mental illness [12–15]. All of these changes and problems may have a significant effect on sleep duration. In addition, the development between regions is unbalanced in China, so significant regional differences of the health outcomes may exist between the urban and the rural and between coastal areas and hinterland.

Based on the data obtained from the National bureau of statistics of China, Guangdong province is the most populous province in China with a population of 113 million in 2018, leading the Gross Domestic Product (GDP) in China for 30 years. Meanwhile, based on the data in 2018, the death rate was higher than the previous 3 years and the proportion of elderly people in Guangdong province were higher than the previous 2 years [16]. Given that the population of Guangdong province is aging rapidly, prevalence rate of chronic disease may comparably increase. Along with the rapid economic growth in Guangdong province, increased psychological stress in adults may lead to increased sleep problems. Prior studies have investigated the relationship between sleep duration and chronic disease in the western countries and a few areas of China [12–15], However, the conditions of sleep duration, mental health and chronic disease in Guangdong province are rarely studied. The association between sleep duration and chronic diseases in the Guangdong population is uncertain.

This study used the cross-sectional data from the Guangdong Provincial Sleep and Psychosomatic Health Survey to investigate whether sleep duration and chronic disease prevalence were associated. To our knowledge, this is the first population-based study conducted with complex stratified sampling method in Guangdong province. We hypothesized that there would be a higher prevalence of chronic disease among short- and long-sleepers, which will also be associated with a few sociodemographic and clinical characteristics.

## Methods

### Sampling and study setting

The study was conducted using data obtained from the Guangdong Provincial Sleep and Psychosomatic Health Survey, which was a public health research project organized and implemented by the Guangdong Provincial Health Commission and the Guangdong Provincial Mental Health Center. The study was approved by the Clinical Research Ethics Committee of Guangdong Provincial People’s Hospital (Reference *No.*GDRE2018230H(R1)). The survey was conducted from January 2019 to October 2019, employing a sampling method involving multistage, stratified, probability-clusters to yield a representative sample of adult population in Guangdong province, China. In the first stage, twenty-one Enumeration Areas of Guangdong province were selected as the primary sampling units with cluster sampling method (Guangzhou, Shenzhen, Zhuhai, Foshan, Zhaoqing, Jiangmen, Huizhou, Dongguan, Zhongshan, Zhanjiang, Maoming, Yangjiang, Yunfu, Shaoguan, Meizhou, Heyuan, Qingyuan, Shantou, Shanwei, Chaozhou, Jieyang). In the second stage, three to four survey districts were selected by using cluster sampling method for each prefecture-level city, and a total of 72 survey districts/counties were selected. In the third stage, two to four neighborhood committees were selected from each selected district/county by simple random method. In the fourth stage, a simple random method was used to select one resident group unit from each selected neighborhood committee, and each resident group unit included 80–200 residents over the age of 18 for investigation. All participants provided informed consent after being informed of study information.

The sample size was calculated using the formula $n= deff\frac{z_a^2\times \mathrm{p}\left(1\hbox{-} \mathrm{p}\right)}{{\mathrm{d}}^2}$. The design effect was assigned as 2.5 and the confidence interval α was 95% of both sides. According to an estimate of the prevalence of depressive disorders in China in 2019, *p* = 6.8% [17]. The allowable error is d = 0.1p. According to the above parameters, the minimum sample size is calculated to be 13,706 people. Considering the response rate was 80%, the planned sample size of this survey was 17,132 people. The survey was completed by 13,768 community-dwelling residents aged 18 years or older, with an effective response rate of 80.42%.

### Procedures

The study was based on each community healthcare center. The methods of combining the centralized survey and household survey were adopted. All of the investigators involved in this study received the required formal training. All the participants were asked questions by investigators face to face. They were also asked to complete the self-reported questionnaires. Trained investigators collected data on sleep duration, basic sociodemographic information, mental health status and history of chronic diseases. Quality controllers checked 100% of the completed questionnaires after each interview. If any logical error or missing data were found, the interviewer and participants were asked to check and clarify.

### Measures

According to recommendations from National Sleep Foundation, for adults aged from 18 to 64, the appropriate sleep duration range is 7 to 9 h per day, and for older adults who are aged over 65, it would be 7 to 8 h per day [18]. 7-9 h of sleep is considered to be most suitable duration for individuals’ mental and physical health [19]. In the survey, all participants were asked about the average number of hours of their sleep each day in the past month. Sleep duration was categorized as three groups: < 7 h, 7–9 h, and ≥ 9 h of sleep per day. The groups of “< 7 h”, “7-9 h”, “≥9 h” were separately called “Short sleeper group”, “Normative sleeper group” and “Long sleeper group” [20].

The sociodemographic variables included gender, age, marital status, education and monthly income. Age was divided into three groups: 18–39, 40–59 and 60–85 years old. Marital status was categorized as single, married and divorced. The educational years are grouped into three categories: less than 9 years, 9–16 years and more than 16 years. Income classes are categorized as 6000 CNY per month and ≥ 6000 CNY per month. Two lifestyle factors including smoking status and drinking status were also included. Besides, body mass index (BMI) was calculated as weight/height^2^ (kg/m^2^) and examined in the study as a metabolic index. It was categorized as < 24 and ≥ 24 (kg/m^2^). Unlike the definition of W.H.O, a BMI ≥24 is considered to be overweight in China [21].

The Patient Health Questionnaire-9 (PHQ9) was used for evaluating depression, which was a widely used questionnaire and was designed to meet the nine criteria used from a diagnosis of DSM-IV depressive disorder. Participants rate the severity of each symptom item over the past 2 weeks on a 4-point Likert-type scale (from 0 to 3). Items can be summed up to generate a continuous measure of depressive symptoms. A cut-point of 10 is used to indicate at least moderate level of depressive symptoms [22]. The Chinese version of PHQ-9 had a sensitivity of 93.33% and a specificity of 96.83% for major depression [23]. The reliability of PHQ-9 was good as well (Cronbach’s alpha = 0.85) [23].

The Generalized Anxiety Disorder-7 (GAD-7) was used for evaluating anxiety, which was a reliable measure to access anxiety symptoms. It also used 4-point Likert type scale. A cut point of 10 is used to indicate at least moderate level of anxiety symptoms [24]. The Chinese version of GAD-7 had a sensitivity of 66.7% and a specificity of 85.8% for generalized anxiety disorder, also with a well reliability (Cronbach’s alpha = 0.90) [25].

To determine the participants’ chronic disease status, they were asked ‘Have you ever been told by a doctor, nurse or other health professional that you have any chronic disease?’ At the same time they were shown a table displaying 22 kinds of chronic diseases, including anemia, arthritis, cerebrovascular disease, coronary heart disease, chronic obstructive pulmonary disease (COPD), diabetes mellitus, gout, hyperlipidemia, hypertension, low back pain, and malignant tumor etc. All the selected chronic diseases were spoken out by investigators to participants in local language (dialect) to help them recall the relevant diagnosis. Participants answered either ‘yes’ or ‘no’. All diseases in this study were classified according to the International Classification of Disease, 10th Revision (ICD-10). The corresponding investigator would confirm the information by checking every participant’s proof of diagnosis or medical records, whenever possible.

### Statistical analysis

We used a complex sampling design data analysis method based on multistage stratified probability-clusters sampling with the SPSS software (version 25.0). All calculations were weighted by gender, age groups, administrative regions according the population data from Guangdong Provincial Bureau of Statistics. The analysis used all participants for whom the variables of interest were available, and missing data were not imputed. The continuous variables were shown by mean value and standard deviation. The categorical variables were shown by frequency and percentage. The comparisons between short, medium and long sleepers by all the characteristic variables were calculated by Rao-Scott Chi-square test. Multinomial logistic regression models were used to explore the correlates of short and long sleep duration. Each parameter was evaluated with univariate and multivariate analysis. A *P* value less than 0.05 was considered statistically significant. In addition, statistical significance does not necessarily imply that the result is important in practice as these tests have a tendency to yield small *p*-values (indicating significance) as the size of the data sets increase, so the practical significance merits further discussion.

## Results

A total of 13,768 individuals were included in the analysis. The average sleep duration was 6.75 ± 1.11 h. Table 1 represented the comparison between short, normative and long sleepers by sociodemographic characteristics and mental health status. Educational level, smoking status, drinking status, BMI, PHQ9 and GAD7 scores were significantly associated with sleep duration.

**Table 1:** Sociodemographic and mental health status of different groups of sleep duration of individuals aged from 18 to 85 years old in Guangdong province in China (*N* = 13,768)

Variables	Short sleeper		Normative sleeper		Long sleeper		Total		X^2^	*P* value
	n	%	n	%	n	%	n	%		
Age									37.06	0.105
18-39y	3661	62.3	5465	61.8	387	63.6	9513	62.0		
40-59y	1645	27.2	2110	28.1	109	21.0	3864	27.6		
60-85y	131	10.5	232	10.1	28	15.4	391	10.4		
Gender									3.04	0.508
Male	2514	53.0	3414	51.7	231	50.2	6159	52.2		
Female	2923	47.0	4393	48.3	293	49.8	7609	47.8		
Educational years									60.40	< 0.001^*^
≤ 9 years	557	14.0	927	15.6	105	26.2	1589	15.4		
9–15 years	2910	54.6	4070	52.9	287	49.7	7267	53.5		
> 16 years	1970	31.3	2810	31.5	132	24.1	4912	31.1		
Marrital status									23.91	0.115
Single	1756	29.7	2493	29.4	144	32.4	4433	29.6		
Married	3537	66.5	5167	68.2	147	65.0	9038	67.4		
Divorced	144	3.8	334	2.4	6	2.6	297	3.0		
Income class (CNY)									7.35	0.115
≤ 6000	4037	75.8	5730	74.6	405	79.2	10,172	75.2		
> 6000	1400	24.2	2077	25.4	119	20.8	3596	24.8		
Smoking status									27.97	0.002^*^
Smoke	1363	29.1	1644	25.0	128	27.2	3135	26.7		
Never smoke	4074	70.9	6163	75.0	396	72.8	10,633	73.3		
Drinking status									42.41	< 0.001^*^
Drink	1061	22.1	1217	17.8	83	16.1	2361	19.4		
Never drink	4376	77.9	6590	82.2	441	83.9	11,407	80.6		
BMI									25.00	0.008^*^
< 24	3929	70.3	6022	74.0	413	75.6	10,364	72.6		
≥ 24	1508	29.7	1785	26.0	111	24.4	3404	27.4		
PHQ9									334.69	< 0.001^*^
No depression	3023	57.7	5521	72.1	399	78.3	8943	66.6		
Have depression	2414	42.3	2286	27.9	125	21.7	4825	33.4		
GAD7									216.71	< 0.001^*^
No anxiety	3434	64.9	5865	75.7	421	82.4	9720	71.7		
Have anxiety	2003	35.1	1942	24.3	103	17.6	4048	28.3		

^*^ *P*-value calculated from Rao-Scott chi-square test for categorical variables. Numbers are unweighted. Percentages are weighted

^*^P < 0.05

Table 2 represented the prevalence rates of chronic disease by sleep duration. Short sleepers had a higher prevalence of chronic diseases, including anemia (6.2%, *p* = 0.024), gout (2.8%, *p* = 0.010), hyperlipidemia (3.9%, *p* = 0.003) and low back pain (5.6%, *p* = 0.020) than other types of sleeper.

**Table 2:** Chronic diseases and multi-morbidity in different sleep duration groups

Variables	Sleep duration						Total		X^2^	*P* value
	Short Sleeper		Normative Sleeper		Long Sleeper					
	n	%	n	%	n	%	n	%		
Anemia	333	6.2	416	4.6	34	6.1	783	5.3	17.42	0.024^*^
Arthritis	198	3.9	193	2.9	17	6.1	408	3.4	23.34	0.060
Cardiovascular disease	64	2.4	67	1.7	3	0.7	134	1.9	12.39	0.254
Cerebrovascular disease	28	0.9	29	0.5	4	0.8	61	0.6	8.38	0.176
COPD	22	0.4	27	0.4	4	0.7	53	0.4	1.43	0.542
Diabetes	107	4.0	126	2.9	9	1.9	242	3.3	14.94	0.252
Gout	128	2.8	116	1.9	3	0.6	247	2.2	19.36	0.010^*^
Hyperlipidemia	186	3.9	162	2.4	7	1.4	355	3.0	29.70	0.003^*^
Hypertension	327	8.4	342	7.2	32	7.3	701	7.7	7.66	0.374
Low back pain	271	5.6	258	3.9	9	2.6	538	4.5	25.90	0.020^*^
Malignant tumor	10	0.2	14	0.2	3	0.6	27	0.2	4.31	0.153

^*^ *P*-value calculated from Rao-Scott chi-square test for categorical variables. Numbers are unweighted. Percentages are weighted. COPD: chronic obstructive pulmonary disease

^*^P < 0.05

Table 3 showed the results from multinomial logistic regression models with odds ratios of the associations of short and long sleep duration with sociodemographic characteristics and mental health factors. The results revealed that lower income earners were likely to have short sleep (OR 1.13, 95%CI 1.01–1.27). Being overweight and having depressive symptoms were obviously risk factors for short sleep (BMI: OR 1.24, 95%CI 1.09–1.41; PHQ9: OR 1.75, 95%CI 1.52–2.02).

**Table 3:** Odds ratios (OR) and 95% confidence intervals of sociodemographic factors and mental health factors in relation to sleep duration

Variables	Short vs Normative sleeper	Short vs Normative sleeper	Long vs Normative sleeper	Long vs Normative sleeper
	Crude OR (95% CI)	Adjusted OR (95% CI)	Crude OR (95% CI)	Adjusted OR (95% CI)
Age				
18-39y	NA	NA	NA	NA
40-59y	1.17 (1.05–1.29)	1.31 (1.17–1.47)	0.79 (0.60–1.02)	0.62 (0.47–0.82)
60-85y	1.05 (0.74–1.49)	1.30 (0.90–1.87)	1.50 (0.85–2.64)	0.96 (0.51–1.82)
Gender				
Male	NA	NA	NA	NA
Female	0.95 (0.85–1.06)	0.96 (0.84–1.09)	1.06 (0.83–1.36)	0.93 (0.66–1.30)
Educational years				
≤ 9 years	0.87 (0.71–1.07)	0.88 (0.72–1.08)	1.79 (1.28–2.52)	1.77 (1.26–2.48)
10–15 years	NA	NA	NA	NA
≥ 16 years	0.97 (0.87–1.07)	0.95 (0.86–1.06)	0.82 (0.61–1.09)	0.83 (0.62–1.11)
Marital status				
Single	1.04 (0.93–1.15)	1.10 (0.98–1.23)	1.16 (0.90–1.48)	1.20 (0.92–1.23)
Married	NA	NA	NA	NA
Divorced	1.61 (1.01–2.54)	1.62 (1.00–2.61)	1.13 (0.41–3.16)	0.89 (0.31–2.54)
Income class (CNY)				
≤ 6000	1.07 (0.95–1.19)	1.13 (1.01–1.27) *	1.30 (1.01–1.68)	1.09 (0.83–1.43)
> 6000	NA	NA	NA	NA
Smoking status				
Ex- or current smokers	1.23 (1.09–1.39) *	1.12 (0.98–1.29)	1.12 (0.86–1.46)	1.10 (0.83–1.47)
Never smoke	NA	NA	NA	NA
Drinking status				
Ex- or current drinkers	1.31 (1.15–1.50) *	1.18 (1.02–1.37) ^*^	0.89 (0.66–1.19)	0.83 (0.61–1.14)
Never drink	NA	NA	NA	NA
BMI				
≥ 24	1.20 (1.06–1.37) ^*^	1.24 (1.09–1.41)^*^	0.92 (0.67–1.27)	0.87 (0.63–1.20)
< 24	NA	NA	NA	NA
PHQ9				
Have depressive symptoms	1.90 (1.70–2.12) ^*^	1.75 (1.52–2.02) ^*^	0.72 (0.54–0.95) ^*^	0.88 (0.61–1.26)
No depressive symptoms	NA	NA	NA	NA
GAD7				
Have anxiety symptoms	1.69 (1.50–1.89) ^*^	1.15 (0.99–1.33)	0.67 (0.50–0.88) ^*^	0.76 (0.53–1.09)
No anxiety symptoms	NA	NA	NA	NA

Note: Univariate regression analysis and multinomial regression analysis were used in statistical analysis. Adjusted for educational level, smoking status, drinking status, BMI, PHQ9 and GAD7 factors

^*^P < 0.05

In Table 4, all the selected chronic diseases were included into analyze. Educational level, smoking status, drinking status, BMI, PHQ9 and GAD7 factors were included as covariates in the adjusted model. The crude and adjusted odds ratios of the associations between chronic diseases and sleep duration were listed. Short sleepers were more likely to have anemia, hyperlipidemia and low back pain (Anemia: OR 1.47, 95%CI 1.16–1.85; Hyperlipidemia: OR 1.53, 95%CI 1.10–2.13; Low back pain: OR 1.42, 95%CI 1.06–1.90). Meanwhile, compared to normative sleepers, long sleepers were more likely to have malignant tumor (OR 3.71, 95%CI 1.01–13.58).

**Table 4:** Odds ratios (OR) and 95% confidence intervals of chronic diseases in relation to sleep duration

Variables	Short vs Normative sleeper	Short vs Normative sleeper	Long vs Normative sleeper	Long vs Normative sleeper
	Crude OR (95% CI)	Adjusted OR (95% CI)	Crude OR (95% CI)	Adjusted OR (95% CI)
Anemia	1.38 (1.09–1.74) ^*^	1.47 (1.16–1.85) ^*^	1.35 (0.78–2.36)	1.23 (0.71–2.13)
Arthritis	1.38 (1.00–1.90) ^*^	1.36 (0.99–1.88)	2.22 (0.95–5.18)	2.01 (0.82–4.95)
Cardiovascular disease	1.41 (0.72–2.78)	1.44 (0.75–2.79)	0.42 (0.12–1.47)	0.29 (0.08–1.10)
Cerebrovascular disease	1.87 (0.84–4.15)	1.82 (0.79–4.17)	1.82 (0.61–5.43)	1.58 (0.50–4.94)
COPD	1.04 (0.56–1.95)	1.04 (0.57–1.90)	1.89 (0.62–5.70)	1.75 (0.56–5.45)
Diabetes	1.39 (0.78–2.47)	1.33 (0.74–2.38)	0.66 (0.29–1.49)	0.53 (0.21–1.37)
Gout	1.51 (1.04–2.21) ^*^	1.39 (0.92–2.09)	0.34 (0.10–1.11)	0.35 (0.11–1.18)
Hyperlipidemia	1.65 (1.19–2.29) ^*^	1.53 (1.10–2.13) ^*^	0.59 (0.19–1.82)	0.64 (0.20–2.01)
Hypertension	1.20 (0.90–1.59)	1.13 (0.85–1.52)	1.03 (0.57–1.86)	0.84 (0.45–1.59)
Low back pain	1.46 (1.09–1.94) ^*^	1.42 (1.06–1.90) ^*^	0.65 (0.24–1.81)	0.54 (0.19–1.48)
Malignant tumor	0.97 (0.39–2.43)	0.95 (0.39–2.34)	3.27 (0.88–12.10) ^*^	3.71 (1.01–13.58) ^*^

Note: COPD: chronic obstructive pulmonary disease. Univariate regression analysis and multinomial regression analysis were used in statistical analysis. Adjusted for educational level, smoking status, drinking status, BMI, PHQ9 and GAD7 factors

^*^P < 0.05

## Discussion

The purpose of this study was to investigate the prevalence and correlation of short and long sleep duration among individuals aged from 18 to 85 years old in Guangdong province in China. To the authors’ knowledge, it is the first study using complex stratified sampling method in southern China, aiming at examining whether sleep duration was associated with sociodemographic characteristics, mental health and chronic diseases. We tested our initial hypothesis and the results supported partially for the hypothesis. People who had lower income, who were ex- or current drinkers, who were overweight, and who had depressive symptoms were at higher possibility of having short sleep duration. Furthermore, short sleepers were more likely to have anemia, hyperlipidemia and low back pain, and long sleepers had close relationship with malignant tumor.

Firstly, seeing from the side of overall length of sleep duration, the average sleep duration of this study was 6.75 h, which was lower than in a similar-scale cross-sectional study in Jilin province in China (7.3 h) [13], a community-based study in Beijing (7.8 h) [26] and a Singapore Chinese study (7.0 h) [27]. Considering multiple factors, spatial regional difference, cultural characteristics, the mean age of sampling population, sampling method and inconsistency of definition of short and long sleep duration were the main reasons of the inconsistency of the results. In the study conducted in Singapore, only elderly people aged more than 60 years old were included and change in sleep was categorized as short sleep duration which was < 6 h, long sleep duration which was > 8 h and stable sleep duration which was 6-8 h [27]. The study [13] conducted in Jilin province in China had similar sample size and sample method comparing to this study. However, the results still showed diversity in distinct areas of the same country. Guangdong province belongs to southern coastal China, while Jilin province locates in northeast China. So Guangdong province is much warmer than Jilin province during most of the time. Diet habit, economy level, urbanization level, and rhythm of life also vary between the two provinces, which may contribute to the difference of the residents’ sleep duration.

Regarding sociodemographic characteristics, low-income earners reported more short sleep in our study. Short sleep duration had been suggested to be positively associated with low income level in western and east Asia studies [28, 29]. In China, a monthly salary below 6000CNY is at relatively low income level. Industrial workers, left-behind farmers, migrant workers, disabled, elderly and so on accounted for the vast majority of low-income earners, which are also vulnerable groups of short sleep. Most of the low-income earners are blue-collar workers, whom have more physical activities, long working time and irregular working rhythm [12]. This may explain the increasing odd ratio of short sleep duration in low-income earners. Income level and educational level are both indicators of socioeconomic position, which is closely associated with living pressure and sleep duration [30]. However, we did not find any differences in education level factor, which was consistent with the Jilin study [13], but inconsistent with some of previous studies in western countries [31, 32]. In developed countries, people who received higher education might have more satisfied employment opportunities supporting higher life quality, whereas those who received lower education may feel stressful each day because of great stress from life and have less sleep time. However, the pattern is different in China, where people with higher education might also have stress in work and in life. As for marital status, no significant results between single, married and divorced samples were found. This suggests that married status have less effect on people’s sleep duration. The results were inconsistent with another study [28], which found single individuals were more likely to report very short sleep. It is possible that single, divorced or married people have similar amount of daily activities. Marriage is becoming decreasingly important for people of all ages.

Unhealthy lifestyle factors contribute certain possibilities to short sleep duration. In our study, associations were found between short sleep and drinking as well as smoking. The first possible reason is that for alcohol users, drinking may mediate their sleep homeostasis through adenosine and the wake-inducing cholinergic neurons in the basal forebrain [33]. For healthy non-alcoholics, although the NREM of the first part of sleep can be promoted and consolidated, the second part of the sleep can be disrupted [33]. And for alcoholics they have problems in controlling drinking, being preoccupied with alcohol, continuing to use alcohol even when it causes problems and having to drink more to get the same effect. So drinking can alter the sleep architecture, lead to insomnia and difficulty maintaining sleep. For the relationship between smoking and sleep duration, significant link between cigarette use and sleep duration has been reported in adolescence [34]. Our study illustrated a link between smoking and short sleep in adults, which added the proof of this relationship in adult age group. Inadequate sleep duration has been particularly linked with substance use, especially alcohol and cigarette. People who were overweight had high risk of short sleep. Weight gain is associated with short sleep duration, which is consistent with a cross-sectional study conducted in Taiwan of China [35]. Given that energy consumption is abnormal in people suffer from obesity, energy balance should be considered into account. Leptin and ghrelin are hormones recognized to have influence on energy balance [36], which play roles in mediating appetite and energy balance [36].

In the adjusted analysis, this study only found that anemia, hyperlipidemia and low back pain were associated with short sleep while malignant tumor were associated with long sleep. Chronic disease and sleep duration are bidirectionally connected with each other [27]. Short sleep can be a consequence of chronic disease, or a marker of chronic disease. In this study, a strong link between short sleep and anemia was found. Consistent with prior findings, sleep duration is associated with lower hemoglobin levels [37]. Fatigue is an important factor mediating the link between short sleep and anemia. Studies have noted that patients with anemia may have fatigue [38, 39], and that fatigue could lead to physical activities decreased directly and affect the circadian rhythms and sleep duration [40]. Consideration of anemia has been recommended as part of the clinical evaluation of patients with insomnia. As for hyperlipidemia, long sleep duration has been reported to be associated with higher risk of hyperlipidemia [41], while some studies observed positive association between short sleep duration and high TG or TC levels [42]. In our study, short sleeping habits may predispose individuals to hyperlipidemia risk. Other metabolic diseases like hypertension and diabetes mellitus have been mentioned to be associated with aberrant sleep duration in previous epidemiologic studies [4, 43]. However, after we adjusted confounders, hypertension and diabetes mellitus have no significant associations with aberrant sleep duration. Individuals with low back pain were at high risk of having short sleep. Insomnia was commonly reported by patients with low back pain [44]. Since pain experience is closely associated with fatigue, we speculate that back pain and fatigue experienced by patients were strong factors for predicting short sleep.

Malignant tumor had positive relationship with long sleep, which was also mentioned in the study conducted in the UK and USA [45]. Malignant tumor is a disease of high complexity. The relationship between long sleep and malignant tumor incidence may vary depending on the course and types of the disease [45]. Interestingly, in terms of cancer subtypes, long sleep duration was found to be associated with increased risk for colorectal cancer and decreased risk for hormone related cancer [46]. Previous results were partly controversial with our results. Up to date, the mechanism which can explain the effect of long sleep duration on risk of malignant tumor is still obscure. Most of the studies found short sleepers had higher risk of having malignant tumor because of the metabolism of melatonin, which can somehow suppress cancer development [47]. Our result was contradictory from the perspective of functions of melatonin. Long sleepers may be possessed of higher melatonin concentration and subsequently have protective effect in hormone-related cancers [46].

Numerous studies have confirmed that chronic disease were risky predictors for short or long sleep duration [48, 49]. The U-shaped relationships between chronic diseases and sleep duration has been investigated in several studies [50, 51], suggesting the importance of sleep health. Furthermore, a U-shaped distribution across short and long sleep duration has also been found for mortality [52]. The mechanism that leads to mortality is not known, and perhaps the co-occurrence of sleep problems (and other problems) may provide clues to pathophysiology [52].

The large sample size and well representative of Guangdong provincial population were the two major strengths of the study. Multiple complex sampling method was the unique feature of method used in this study. The surveys were conducted by experienced and trained investigators. However, several limitations should be clearly mentioned in this study. First, the study sample only covered the adult population of Guangdong province in China so that the results may not represent the general population of China. Second, participants may misunderstand the definition of actual sleep duration and time of lying in bed. And without objective sleep measurement, the self-reported sleep duration may not represent the real situation. Future studies are warranted to investigate changes to multiple aspects of sleep with objective assessments. Third, the study was designed as a cross-sectional study, and thus the causation of chronic disease and sleep duration could not be examined. Furthermore, other relevant sleep variables were not included into analysis, such as use of hypnotic medication, treatments for specific chronic disease, living conditions, night shift work, cultural factors and so on. These variables may have confounded the results in this study. Another limitation is that we did not exclude other kinds of sleep disorder, such as sleep apnea associated with high BMI or diabetes, restless legs syndrome associated with anemia, sleep walking disorder associated with epilepsy and so on. Other sleep variables, such as sleep quality, daytime functions and sleep efficiency, were not included to measure the sleep condition of participants in this study. In addition, specific information about subtypes of malignant tumor was not collected at the beginning of the study.

Future studies are warranted to investigate changes to multiple aspects of sleep with objective assessments. Further multidimensional studies are also needed to assess the mediating role of mental health in the association between chronic disease and sleep.

## Conclusions

This study included representative and large sample of Guangdong province, and found sociodemographic characteristics were associated with potential risk for short and long sleep duration. Anemia, hyperlipidemia, low back pain and malignant tumor, were confirmed associated with short and/or long sleep duration. Longitudinal studies are needed to gain a better understanding of possible associations, and to figure out the causations between chronic diseases and sleep duration.

## Data Availability

The datasets used and/or analyzed during the current study are available from the corresponding author on reasonable request.

## Abbreviations

BMI: Body mass index
COPD: Chronic obstructive pulmonary disease
GAD-7: Generalized Anxiety Disorder
HPA: The hypothalamic-pituitary-adrenal
PHQ-9: Patient Health Questionnaire-9
REM: Rapid Eye Movement
NREM: Non-rapid Eye Movement
